# TNF-α Predicts Endothelial Function and Number of CD34^+^ Cells after Stimulation with G-CSF in Patients with Advanced Heart Failure

**DOI:** 10.3390/jcdd9080281

**Published:** 2022-08-20

**Authors:** Sabina Ugovšek, Andreja Rehberger Likozar, Sanjo Finderle, Gregor Poglajen, Renata Okrajšek, Bojan Vrtovec, Miran Šebeštjen

**Affiliations:** 1 Faculty of Medicine, University of Ljubljana, 1000 Ljubljana, Slovenia; 2 Department of Vascular Diseases, University Medical Centre Ljubljana, 1000 Ljubljana, Slovenia; 3 Department of Cardiology, University Medical Centre Ljubljana, 1000 Ljubljana, Slovenia

**Keywords:** heart failure, CD34^+^ cells, TNF-α, endothelial function

## Abstract

Patients with advanced heart failure (HF) have reduced cardiac output and impaired peripheral blood flow, which diminishes endothelial shear stress and consequently flow-mediated dilatation (FMD). The aim of our study was to find out whether endothelial dysfunction is associated with the number of CD34^+^ cells and TNF-α levels in patients with ischemic and non-ischemic HF after stimulation with granulocyte colony-stimulating factor (G-CSF). We included 56 patients with advanced HF (LVEF < 35%). Eighteen patients (32.14%) had ischemic and 38 (67.86%) patients had non-ischemic HF. FMD of the brachial artery was performed before the patients underwent 5-day bone marrow stimulation with daily subcutaneous injections of G-CSF (5 μg/kg bid). On the fifth day peripheral blood CD34^+^ cell count was measured. No statistically significant differences were found between the patient groups in NT-proBNP levels ((1575 (425–2439) vs. 1273 (225–2239)) pg/mL; *p* = 0.40), peripheral blood CD34^+^ cell count ((67.54 ± 102.32 vs. 89.76 ± 71.21) × 10^6^; *p* = 0.32), TNF-α ((8.72 ± 10.30 vs. 4.96 ± 6.16) ng/mL; *p* = 0.13) and FMD (6.7 ± 5.4 vs. 7.2 ± 5.9%; *p* = 0.76). In a linear regression model, only FMD (*p* = 0.001) and TNF-α (*p* = 0.003) emerged as statistically significant predictors of CD34^+^ cells counts. Our study suggests that TNF-α is a good predictor of impaired endothelial function and of CD34^+^ cells mobilization after G-CSF stimulation in patients with advanced HF of ischemic and non-ischemic origin.

## 1. Introduction

Endothelial function is impaired in patients with coronary artery disease, acute and chronic heart failure and in patients with atherosclerotic risk factors and no clinically evident heart disease [1,2,3,4,5,6]. Reduced endothelial function is due to decreased endothelial production and/or increased degradation of nitric oxide (NO) [7]. Since NO is thought to represent an anti-atherosclerotic molecule, the prognostic impact of endothelial dysfunction on vascular events has been previously studied in conditions known to represent risk factors for atherosclerotic disease. Indeed, endothelial dysfunction is independently associated with increased incidence of hospitalization, cardiac transplantation, or death in patients with chronic heart failure (HF) of ischemic and non-ischemic origin [8,9]. In HF, nitric oxide production is diminished, whereas rate of endothelial apoptosis is increased. Endothelial progenitor cells (EPCs) have been isolated from circulating CD34^+^ mononuclear cells and are thought to participate in vasculogenesis [10]. CD34 is a transmembrane phosphoglycoprotein, found on hematopoietic stem and on mature endothelial cells [11,12]. Furthermore, hematopoietic stem cells express CD133+, which is not found on mature endothelial cells [11]. To differentiate between the two, CD133+ typing is needed [11]. Clinically, CD34 is associated with the selection and enrichment of hematopoietic stem cells for bone marrow transplants. The CD34^+^ cells represent a small proportion of the total cell population and also indicate a distinct subset of cells with enhanced progenitor activity. CD34 is not a specific marker as it is present on diverse progenitors of nonhematopoietic cell types including muscle satellite cells, corneal keratocytes, interstitial cells, epithelial progenitors, and vascular endothelial progenitors [12]. Tumor necrosis factor-α (TNF-α) is a potent and reversible inhibitor of colony formation of human CD34^+^ bone marrow cells stimulated by granulocyte colony-stimulating factor (G-CSF) [13].

It is also known that the number of circulating CD34^+^ cells is decreased in patients with chronic HF and that their number decreases with increased New York Heart Association (NYHA) class [10]. Bone marrow exhaustion, impaired mobilization and increased apoptosis of progenitor cells have all been suggested to account for this decrease [14,15,16]. Valgimigli et al. reported that the aetiology of advanced HF does not play a role in the number of circulating CD34^+^ cells [10]. On the other hand, Theiss et al. showed that patients with idiopathic dilatative cardiomyopathy have a greater number of CD34^+^ cells than patients with ischemic cardiomyopathy [15]. In a study of Kakzanov et al., no difference in number of CD34^+^ cells was found in patients with HF with reduced ejection fraction (HFrEF) and in patients with HF with preserved ejection fraction (HFpEF), despite the fact that in a group of patients with HFrEF significantly more patients had ischemic heart disease [17]. The number of transplanted stem cells is one of the most important factors for successful treatment of HF [18,19,20,21].

TNF-α, one of the main atherogenic cytokines, has a negative effect on both endothelial function and CD34^+^ cell counts. TNF-α selectively inhibits receptor-mediated release of NO from endothelial cells without affecting its basal release. TNF-α-induced suppression of endothelial NO release is due to downregulation of mRNA for constitutive NO synthase [22]. Among cytokines and neurohormonal activators, only TNF-α showed to be an independent predictor of decreased endothelial function, measured as flow-mediated dilation (FMD) of the brachial artery in patients with idiopathic dilated cardiomyopathy [23]. TNF-α is also a potent and reversible inhibitor of colony formation of human CD34^+^ bone marrow cells stimulated by G-CSF [13]. On the other hand, patients with HF have been shown to have a significantly increased concentration of TNF-α compared to their healthy peers. In the same study, the concentration of TNF-α increased with the NYHA class [24].

The aim of our study was to find out whether endothelial dysfunction is associated with the number of CD34^+^ cells and TNF-α levels in patients with advanced HF of ischemic and non-ischemic origin after stimulation with G-CSF.

## 2. Methods

### 2.1. Patients

A subset of patients in our prospective study were included from our previous study [25]. Briefly, 56 consecutive patients with advanced HF regardless of the HF aetiology, and eligible for CD34^+^ cell therapy, were included. All patients underwent 5-day bone marrow stimulation with daily subcutaneous injections of G-CSF (5 μg/kg bid) before transendocardial transplantation of CD34^+^ cells as described previously [25].

Subjects between ages of 18 and 66 years with advanced HF of ischemic and non-ischemic aetiology, in NYHA function class II-IV, and left ventricular ejection fraction (LVEF) <35%, were eligible for the study. The subjects also had to receive optimal medical therapy for at least 1 year and had to be hospitalized for minimum of two times in the last year. The female subjects were all after menopause. The exclusion criteria were malignant or hematological disease in the last 5 years.

The protocol was approved by the National Medical Ethics Committee of the Republic of Slovenia (reference number: KME 136/03/11, date of approval: 15 March 2011). All subjects provided written informed consent prior to the study. All procedures followed institutional guidelines.

### 2.2. Study Design

The patients’ blood samples were taken at the beginning of the study (day 0). The patients then underwent 5-day bone marrow stimulation with daily subcutaneous injections of G-CSF (5 μg/kg bid). On the fifth day peripheral blood CD34^+^ cells were collected with the Amicus cell separator (Baxter Healthcare, Deerfield, IL, USA) and counted.

Before the stimulation with G-CSF ultrasound measurement of flow-mediated dilatation (FMD) of the brachial artery and transthoracic echocardiography were performed. The patients also completed a 6-min walk test.

### 2.3. Blood Samples

The subjects’ blood was drawn from the median cubital vein after a 12-h fast and 15-min of rest in supine position on day 0. The blood for hematological analyses was collected into 3 mL tubes coated with ethylenediaminetetraacetic acid (EDTA) (Vacutainer^TM^, Becton and Dickinson, Plymouth, England) and centrifuged at 4500 rpm at 20 °C for 10 min.

A 3 mL EDTA-tube containing aprotinin was used for determining N-terminal prohormone of brain natriuretic peptide (NT-proBNP) and TNF-α. These samples were immediately placed on ice for up to 4 h, and then centrifuged at 4500 rpm for 15 min at 0 °C. The serum was extracted and stored at 80 °C until NT-proBNP and TNF- α measurement.

### 2.4. Blood Analyses

All analyses were performed at a central independent laboratory, blinded to the clinical data. Whole blood samples were used for the following analyses: red blood cells count (RBC), white blood cells count (WBC), hemoglobin, platelet count, red blood cell distribution width (RDW). The analysis was performed on automated blood count using Cobas Minos STEX^®^ analyzer (Roche, Basel, Switzerland). Biochemical analysis was performed on Ektachem 250 Analyzer^®^ (Estman Kodak Company, Rochester, NY, USA). NT-proBNP assays were performed using a commercially available kit (Roche Diagnostics, Mannheim, Germany). Enzyme-linked immunosorbent assay (ELISA) was used to measure the serum levels TNF-α (Quantikine(r) HS Human TNF-a Immunoassay), according to the instructions of the manufacturer (R&D Systems, Minneapolis, MN, USA).

### 2.5. Immunomagnetic Positive Selection of CD34^+^ Cells and Cell Counting

The magnetic cell separator Isolex 300i (Nexell Therapeutics INC, Irvine, CA, USA) was used for the immunomagnetic positive selection of CD34^+^ cells. In the closed system, the collected cells were washed to remove the platelets, sensitized with mouse monoclonal anti-CD34 antibodies and then incubated with immunomagnetic beads coated with polyclonal sheep anti-mouse antibodies (Dynabeads-Dynal AS, Oslo, Norway). The bead/CD34^+^ cell rosettes were separated in the magnetic field from other cells and CD34^+^ cells were released from the Dynabeads using an octapeptide with an affinity for anti-CD34 antibodies. The cells were then counted using a flow cytometer (Beckman Coulter Inc., Brea, CA, USA).

### 2.6. 6-min Walk Test

A 6-min walk test was performed by a blinded observer according to the standard clinical protocol [26].

### 2.7. Brachial Artery Ultrasound Imaging

All subjects were studied following a 12-h fast in a room with room temperature 22–26 °C. Ultrasound measurements were performed by an independent observer using HDI 5000 Ultrasound system (Advanced Technology Laboratories, Seattle, WA, USA). The brachial artery was visualized using a high-resolution 7-MHz linear array ultrasound transducer. Before the beginning of the study, we measured the subjects’ blood pressure after 10 min of rest in sitting position. Three consecutive measurements taken every 2 min were averaged. The patients then had to rest for 30 min in supine position. During the examination ECG was monitored. All measurements were performed at the end of the diastole.

A 5 cm-wide blood pressure cuff was placed around the lower forearm. Measurements were performed from 2 to 10 cm proximal to the antecubital fossa. Vessel diameter was imaged in the longitudinal view as the widest diameter between the superior and inferior intimal markings of the vessel. Blood flow velocity was recorded at rest. The brachial artery was then occluded with rapid inflation of the cuff to 30 mmHg above systolic pressure for 4.5 min. The blood velocity was measured 15 to 20 s after the release of the occlusion; vessel diameter was measured 60 to 90 s after the release. Flow-mediated dilatation was determined as the percent change in brachial artery diameter after cuff release compared with the resting baseline brachial artery diameter.

After 15 min of rest we measured the endothelium independent dilatation of the brachial artery (GTN). We used 0.4 mg of glyceryl trinitrate applied sublingually. Again, endothelium independent dilatation was calculated as the percent change in brachial artery diameter compared with the resting baseline brachial artery diameter.

### 2.8. Transthoracic Echocardiography

The echocardiography data were recorded and analyzed by an independent echocardiographer who was blinded both for randomization and timing of the recordings. The studies were conducted using the Vivid q ultrasound (GE medical systems, Israel). Standard two- and four-chamber views were obtained from apical transducer position. The biplane Simpson’s method was used to calculate the left ventricular ejection fraction. All measurements were performed in triplicates from three consecutive cardiac cycles.

### 2.9. Statistical Analysis

Baseline clinical characteristics of the study population are expressed as mean ± standard deviation or median (interquartile range), if the parameters were not normally distributed. An independent-samples *t*-test or Welch *t*-test was performed for parameters that were normally distributed, otherwise Mann–Whitney U test was used. A chi-square test was performed between the ischemic and non-ischemic HF group and different drugs taken by the subjects. If one or more expected cell frequencies was less than five, the Fisher’s exact test was used. The correlation between parameters was calculated using Spearman rank-order correlation. General linear model analyses using age, ejection fraction, pro-BNP, and platelets as covariates were performed to test the influence of the FMD and TNF-α on the CD34^+^ cell counts. A *p*-value less than 0.05 was considered statistically significant. All analyses were performed using Statistica ver. 7.1 software (StatSoft Inc., 2005, Tulsa, OK, USA).

## 3. Results

### 3.1. Patients Clinical and Laboratory Characteristics

**Figure 1:**
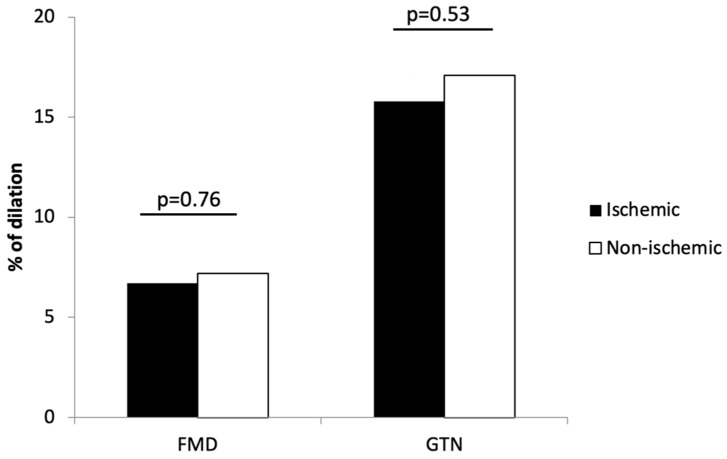
Endothelial dependent (FMD) and endothelial independent (GTN) dilatation of brachial artery in patients with ischemic and non-ischemic HF. Shown are means. No statistically significant differences were found between the two groups of patients (*p* > 0.05).

There were 18 subjects in the group with ischemic HF and 38 subjects in the group with non-ischemic HF. Their characteristics are shown in Table 1. Patients with ischemic HF were older than patients with non-ischemic HF (58.00 ± 5.89 vs. 52.39 ± 10.40 years; *p* = 0.01), had lower total serum cholesterol (3.56 ± 0.66 vs. 4.70 ± 1.21 mmol/L; *p* ≤ 0.001), LDL-cholesterol (1.96 ± 0.50 vs. 2.85 ± 0.73 mmol/L; *p* ≤ 0.001) and HDL-cholesterol (0.97 ± 0.20 vs. 1.20 ± 0.40 mmol/L; *p* = 0.01). A statistically significant difference was not observed between the groups in NT-proBNP serum concentration levels ((1575 (425–2439) vs. 1273 (225–2239) pg/mL); *p* = 0.40), peripheral blood CD34^+^ cell count after G-CSF stimulation ((67.54 ± 102.32 vs. 89.76 ± 71.21) × 10^6^); *p* = 0.32) and TNF-α ((8.72 ± 10.30) vs. (4.96 ± 6.16) ng/mL); *p* = 0.13). Figure 1 shows endothelium-dependent (FMD) (6.7 ± 5.4 vs. 7.2 ± 5.9%; *p* = 0.76) and endothelium-independent dilatation (GTN) (15.8 ± 7.1 vs. 17.1 ± 7.8; *p* = 0.53) of the brachial artery, which did not differ between the two groups of patients. No differences were found between the studied groups regarding treatment with beta-blockers, angiotensin-converting-enzyme (ACE) inhibitors or angiotensin receptor blockers (ARBs), aldosterone antagonists or diuretics. Patients with ischemic HF were more frequently treated with statins (*p* ≤ 0.001) and antiplatelet agents (*p* ≤ 0.001).

### 3.2. Flow-Mediated Dilation and CD34^+^ Cell Counts Predictors

The relationships between FMD and various parameters in patients with ischemic and non-ischemic aetiology are presented in Table 2. In 18 patients with ischemic HF significant correlations between FMD and the number of peripheral blood CD34^+^ stem cells were found after G-CSF stimulation, (ρ = 0.62, *p* = 0.01), total cholesterol (ρ = 0.66, *p* = 0.003), HDL cholesterol (ρ = 0.57, *p* = 0.01) and LDL cholesterol (ρ = 0.56, *p* = 0.01). On the other hand, significant positive correlations between FMD and the number of CD34^+^ stem cells (ρ = 0.45, *p* = 0.004) and platelet count (ρ = 0.38, *p* = 0.02) were found in 38 patients with non-ischemic HF after stimulation with G-CSF.

**Figure 2:**
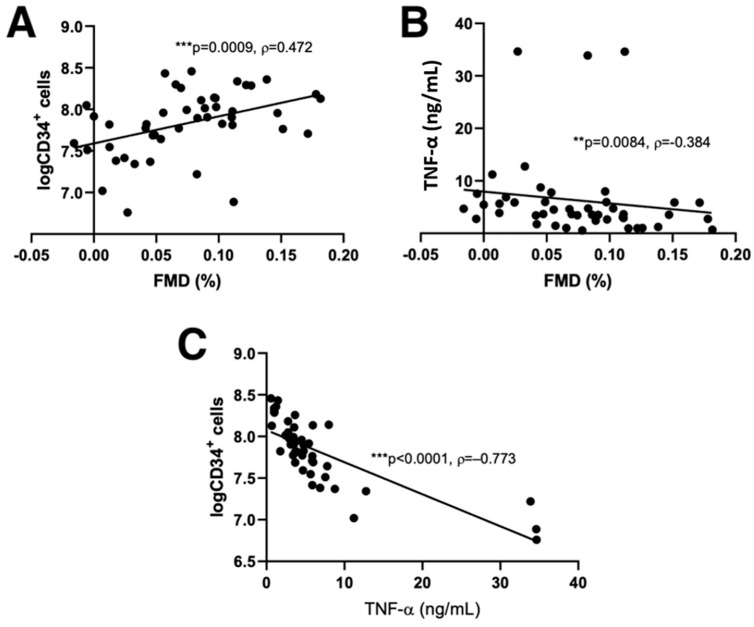
Significant correlations between FMD, number of CD34^+^ cell counts after stimulation and TNF-α. Significant correlations were found between FMD and CD34^+^ cell counts (**A**), TNF-α (**B**), and between CD34^+^ cell counts and TNF-α (**C**). Scatter plots are shown with the results of Spearman correlation analysis (** *p* < 0.01; *** *p* < 0.001; ρ, Spearman’s correlation coefficient). FMD (flow-mediated dilatation), TNF-α (tumor necrosis factor-α).

Combining both groups of patients, we found significant correlations between FMD and blood glucose levels (ρ = −0.32; *p* = 0.02), platelet count (ρ = 0.40; *p* = 0.002), TNF-α levels (ρ = 0.384; *p* = 0.0084) and the number of CD34^+^ cells after G-CSF stimulation (ρ = 0.51; *p* ≤ 0.001). TNF-α was significantly associated with the number of CD34^+^ cells and both parameters were also significantly associated with FMD (Figure 2). In a linear regression model (Table 3), explaining 43% of variability (*p* = 0.005), FMD (*p* = 0.001) and TNF-α (*p* = 0.003) emerged as statistically important predictors of the number of CD34^+^ cells after G-CSF stimulation.

## 4. Discussion

To the best of our knowledge, this is the first study to examine the association between the number of peripheral blood CD34^+^ stem cells after 5-day bone marrow stimulation with daily subcutaneous injections of G-CSF (5 μg/kg bid), TNF-α and endothelial function, measured as FMD of brachial artery in patients with advanced HF. In multiple regression models, both FMD and TNF-α emerged as statistically significant predictors of the number of CD34^+^ stem cells after G-CSF stimulation in patients with HF of ischemic and non-ischemic aetiology as well as in the combined study group regardless of aetiology.

Previous studies examined the number of CD34^+^ cells in basal conditions, while in our study the number of CD34^+^ cells was measured after stimulation with G-CSF. TNF-α inhibits the generation of CD34^+^ cells both in basal conditions through the inhibition of key regulator of haematopoiesis stem cell factor (SCF), as well as upon stimulation with G-CFS [13]. Hence, our results could be compared to the results of the studies where the number of CD34^+^ cells under basal conditions was determined. Given that we did not find any statistically significant differences in the number of CD34^+^ cells between patients with ischemic and non-ischemic HF, we can assume that there are no differences in the functional capacity of the bone marrow between the two studied groups. In the study of Valgimigli et al., CD34^+^ were increased in patients in NYHA class I-III, but decreased in class IV compared even to control subjects [10]. They found inverse correlation between the number of CD34^+^ cells and TNF-α and related soluble receptors. Their conclusion was that during the early stages of the disease, when TNF-α is not yet significantly elevated, CD34^+^ are increased as a reflection of a functional bone marrow response to diffuse and severe endothelial damage. In advanced HF functional bone marrow response is overwhelmed by suppressive effect of TNF-α, which increases significantly in advanced HF. Theiss et al. found no correlations between mobilization of CD34^+^ cells, levels of TNF-α and NYHA class [15]. This finding may be due to the fact that they included patients in class II and III only, and excluded patients with vascular disorders, myocarditis, congenital diseases and heart disease due to hypertension. In our group of patients with non-ischemic HF, patients with idiopathic dilative cardiomyopathy, after myocarditis, as well as heart disease due to hypertension were included.

In the Multi-Ethnic Study of Atherosclerosis (MESA) study, FMD was shown to be a good predictor of future HF of ischemic aetiology or HF with decreased ejection fraction, but not for idiopathic dilated cardiomyopathy or HF with preserved ejection fraction [27]. In a study of Shah et al., which included 11 patients with ischemic and 12 patients with non-ischemic heart failure, it was found that FMD was impaired only in the first group, while in patients with non-ischemic HF it was comparable to the control group [28]. In our study, the patient groups according to the aetiology of HF did not differ in terms of FMD, CD34^+^ cell count, or TNF-α levels. Nevertheless, since FMD of the brachial artery is a very good predictor of endothelial function of coronary arteries, we could assume that endothelial function of coronary arteries is impaired equally in patients with ischemic and non-ischemic HF [29]. Myocardial perfusion was found to correlate with FMD in patients with non-obstructive coronary artery disease, but such correlation was not found confirmed in patients with non-ischemic HF [30,31]. In the latter study abnormal myocardial perfusion did characterize patients with non-ischemic HF, but did not correlate to FMD [31]. It is interesting that FMD was not impaired compared to healthy volunteers, contrary to some other studies in patients with non-ischemic HF [32,33]. Stolen et al. justified their findings by the fact that their healthy volunteers were sedentary and overweight [31]. Therefore, it is tempting to speculate that in both ischemic and non-ischemic HF, myocardial perfusion is abnormal. Unfortunately all the above-mentioned studies [27,28,29,30,31,32,33] do not possess data on TNF-α levels. Hence, our study is the first to show the correlation between FMD and TNF-α levels and suggests further investigation.

Oikonomou et al. found that treatment with atorvastatin in patients with ischemic HF mobilizes and increases the number of circulating endothelial progenitor cells (EPCs), including CD34^+^, in peripheral blood [34]. This increase was dose-dependent and not affected by baseline cholesterol levels. Similar to the increase in the number of EPCs, they also observed the increase of FMD and decrease of TNF-α levels. Statins improve endothelial function with increased production and decreased degradation of NO, a principle regulator of endothelial function, through different mechanisms [35]. In patients with HF, statin therapy was also shown to decrease TNF-α, one of the most important regulators for both FMD and number of circulating CD34^+^ cells [36]. In our study, more patients with ischemic HF were treated with statins compared to non-ischemic HF patients, but despite that we found no difference in endothelial function between the two groups of patients.

Statins improve endothelial function in patients with coronary artery disease without HF, and in patients with ischemic HF [37,38]. In studies where patients with ischemic and non-ischemic HF were included, statin treatment also improved FMD [39,40], but there is no study examining the effect of statins in patients with non-ischemic HF. More frequent statin therapy in the group of patients with ischemic HF might be the reason for the difference in correlations between FMD and the number of CD34^+^ cells in patients with ischemic and non-ischemic HF in our study. This is in contrast to the finding of Tousoulis et al., who, despite the improvement of FMD and increase of CD34^+^ cells after treatment with rosuvastatin in patients with ischemic and non-ischemic HF, found no correlations between these two parameters [41].

Our study is limited by a relatively small study group, in particular the number of patients in the ischemic HF group as well as heterogeneous population in the non-ischemic HF group. This is due to the design of the study as the patients with advanced HF regardless of the HF aetiology, and eligible for CD34^+^ cell therapy, were included in a consecutive manner. However, the number of patients included in our study is comparable to previous studies investigating CD34^+^ cells [15,17]. Another limitation of our study is the absence of CD133^+^ typing due to found restriction. CD133+ typing is needed to differentiate between hematopoietic stem cells and mature endothelial cells [11]. Although, in our study, CD34^+^ cells, regardless of the source, were decreased in the presence of higher TNF-α levels.

The main difference between our study and previous studies investigating CD34^+^ cells is that we measured the number of CD34^+^ cells after 5-day bone marrow stimulation with daily subcutaneous injections of G-CSF (5 μg/kg BID), while previous studies measured the number of cells in basal conditions [10,15]. Unfortunately, we do not have data on the baseline CD34^+^ cell counts. Therefore, we obtained only an absolute number of cells after bone marrow stimulation with G-CSF and not a relative increase of the number of CD34^+^ cells, which would probably show the potential of bone marrow to increase the CD34^+^ production. Nevertheless, for patients with HF undergoing stem cells transplantation, the most important factor for a positive outcome is the amount of CD34^+^ cells in patients with ischemic, as well as in non-ischemic, HF [19,20,21].

Considering that in our study, increased levels of TNF-α were associated with impaired endothelial function measured by FMD, as well as with a lower number of CD34^+^ cells after stimulation with G-CSF, we suggest that high TNF-α levels are one of the main factors affecting both the endothelium and the bone marrow function in patients with HF regardless of the cause of HF.

## Figures and Tables

**Table 1:** Clinical and laboratory values in patients with ischemic and non-ischemic HF.

Variable (Unit)	Ischemic (n = 18)	Non-Ischemic (n = 38)	*p*
**Age (years)**	**58.00 ± 5.89**	**52.39 ± 10.40**	**0.01**
LVEF (%)	27.28 ± 4.45	29.67 ± 4.13	0.08
Na^+^ (mmol/L)	141 ± 3	141 ± 3	0.34
K^+^ (mmol/L)	4.5 ± 0.4	4.6 ± 0.4	0.91
BUN (mmol/L)	8.15 ± 2.9	6.85± 4.1	0.40
Creatinine (μmol/L)	92.94 ± 24.41	91.58 ± 23.02	0.84
**Total cholesterol (mmol/L)**	**3.56 ± 0.66**	**4.70 ± 1.21**	**<0.001**
**LDL cholesterol (mmol/L)**	**1.96 ± 0.50**	**2.85 ± 0.73**	**<0.001**
**HDL cholesterol (mmol/L)**	**0.97 ± 0.20**	**1.20 ± 0.40**	**0.01**
Triglycerides (mmol/L)	1.50 ± 1.0	1.42 ± 1.2	0.99
NT-proBNP (pg/mL)	1575 (425–2439)	1273 (225–2239)	0.40
6-min walk test (m)	417.61 ± 105.46	460.80 ± 109.69	0.17
WBC (×10^9^/L)	7.25 ± 1.73	7.22 ± 1.57	0.95
RBC (×10^12^/L)	4.77 ± 0.38	4.79 ± 0.77	0.72
Hb (g/L)	146.39 ± 12.44	145.79 ± 10.42	0.85
Platelets (×10^9^/L)	195.00 ± 48.47	218.55 ± 47.30	0.09
RDW (%)	14.68 ± 1.48	14.23 ± 1.08	0.21
Blood glucose (mmol/L)	5.65 ±2.8	5.4± 1.6	0.48
CD34^+^ cells (×10^6^)	67.54 ± 102.32	89.76 ± 71.21	0.32
TNF-α (ng/mL)	8.72 ± 10.30	4.96 ± 6.16	0.13
**ASA**	**14/18 (77.8%)**	**6/38 (15.8%)**	**<0.001**
β-blocker	18/18 (100.0%)	37/38 (97.4%)	0.99
ACE inhibitor/ARBs	18/18 (100.0%)	36/38 (94.7%)	0.99
**Statins**	**17/18 (94.4%)**	**14/38 (36.8%)**	**<0.001**
Aldosterone inhibitors	15/18 (83.3%)	23/38 (84.2%)	0.99
Furosemide	11/18 (61.1%)	23/38 (60.5%)	0.97
Ivabradine	1/18 (5.6%)	4/38 (10.5%)	0.99
Digoxin	1/18 (5.6%)	6/38 (15.8%)	0.41

LVEF (left ventricular ejection fraction), Na^+^ (sodium), K^+^ (potassium), BUN (blood urea nitrogen), LDL (low-density lipoprotein), HDL (high-density lipoprotein), NT-proBNP (N-terminal prohormone of brain natriuretic peptide), WBC (white blood cells), RBC (red blood cells), Hb (hemoglobin), RDW (red blood cell distribution width), ASA (acetylsalicylic acid), ACE (angiotensin-converting-enzyme), ARBs (angiotensin receptor blockers), TNF-α (tumor necrosis factor-α). Shown are means ± standard deviation or medians (interquartile range). Parameters where statistically significant differences between the studied groups were found are shown in bold (*p* < 0.05).

**Table 2:** Results of correlation analysis between FMD and clinical and laboratory parameters in patients with ischemic and non-ischemic HF.

	Ischemic HF (n = 18)		Non-Ischemic HF (n = 38)	
Variable	ρ	*p*	ρ	*p*
Age	0.01	0.98	−0.08	0.64
LVEF	0.36	0.14	0.24	0.14
Na^+^	0.21	0.40	0.17	0.32
K^+^	0.01	0.96	−0.22	0.19
BUN	−0.16	0.53	−0.20	0.22
Creatinine	−0.28	0.25	−0.08	0.64
Total cholesterol	**0.66**	**0.003**	0.01	0.96
LDL	**0.56**	**0.01**	0.04	0.79
HDL	**0.57**	**0.01**	−0.02	0.92
Triglycerides	0.30	0.22	−0.19	0.24
NT-proBNP	−0.17	0.51	0.06	0.73
6-min walk test	0.34	0.16	0.02	0.92
WBC	−0.18	0.47	0.03	0.87
RBC	−0.12	0.62	0.10	0.56
Hb	−0.10	0.70	−0.04	0.82
Platelets	0.36	0.14	**0.38**	**0.02**
RDW	0.12	0.64	0.24	0.15
Blood glucose	−0.26	0.29	−0.30	0.06
**CD34^+^ cells**	**0.62**	**0.01**	**0.45**	**0.004**
**TNF-** **α**	**0.54**	**0.02**	**0.57**	**0.001**

ρ (Spearman correlation coefficient), LVEF (left ventricular ejection fraction), Na^+^ (sodium), K^+^ (potassium), BUN (blood urea nitrogen), LDL (low-density lipoprotein), HDL (high-density lipoprotein), NT-proBNP (N-terminal prohormone of brain natriuretic peptide), WBC (white blood cells), RBC (red blood cells), Hb (haemoglobin), RDW (red blood cell distribution width), TNF-α (tumor necrosis factor-α). Parameters where statistically significant differences between the studied groups were found are shown in bold (*p* < 0.05).

**Table 3:** Linear regression analysis explaining the role of various risk factors in the number of CD34^+^ cells (*p* = 0.004, R^2^ = 0.43).

Variable	B	SE_B_	β	*p*
Intercept	254.343	92.312		0.002
Age	−1.321	1.226	−0.153	0.39
EF	0.783	1.1215	0.054	0.52
NT-proBNP	−7.234	2.453	−0.223	0.14
Platelets	−0.234	0.263	−0.176	0.52
Ischemic heart failure	0.543	1.235	0.456	0.62
**FMD**	**597.145**	**230.654**	**0.543**	**0.001**
**TNF-** **α**	**−392.432**	**187.480**	**−0.654**	**0.003**

B = unstandardized regression coefficient, SE_B_ = standard error of the coefficient, β = standardized coefficient, EF (ejection fraction), FMD (flow-mediated dilatation of brachial artery), TNF-α (tumor necrosis factor-α). Variables with statistically significant contribution are shown in bold (*p* < 0.05).

## Data Availability

The data presented in this study are available on request from the corresponding author. The data are not publicly available due to protection of the privacy of personal data.
